# The inner membrane protein YhiM links copper and CpxAR envelope stress responses in uropathogenic *E. coli*

**DOI:** 10.1128/mbio.03522-23

**Published:** 2024-03-12

**Authors:** Panatda Saenkham-Huntsinger, Matthew Ritter, George L. Donati, Angela M. Mitchell, Sargurunathan Subashchandrabose

**Affiliations:** 1 Department of Veterinary Pathobiology, College of Veterinary Medicine and Biomedical Sciences, Texas A&M University, College Station, Texas, USA; 2 Department of Chemistry, Wake Forest University, Winston-Salem, North Carolina, USA; 3 Department of Biology, College of Science, Texas A&M University, College Station, Texas, USA; The Ohio State University School of Medicine, Columbus, Ohio, USA

**Keywords:** *E. coli*, UPEC, YhiM, copper, CpxAR, envelope stress

## Abstract

Urinary tract infection (UTI) is a ubiquitous infectious condition, and uropathogenic *Escherichia coli* (UPEC) is the predominant causative agent of UTI. Copper (Cu) is implicated in innate immunity, including against UPEC. Cu is a trace element utilized as a co-factor, but excess Cu is toxic due to mismetalation of non-cognate proteins. *E. coli* precisely regulates Cu homeostasis via efflux systems. However, Cu import mechanisms into the bacterial cell are not clear. We hypothesized that Cu import defective mutants would exhibit increased resistance to Cu. This hypothesis was tested in a forward genetic screen with transposon (Tn*5*) insertion mutants in UPEC strain CFT073, and we identified 32 unique Cu-resistant mutants. Transposon and defined mutants lacking *yhiM*, which encodes a hypothetical inner membrane protein, were more resistant to Cu than parental strain. Loss of YhiM led to decreased cellular Cu content and increased expression of *copA*, encoding a Cu efflux pump. The CpxAR envelope stress response system was activated in the *ΔyhiM* mutant as indicated by increased expression of *cpxP*. Transcription of *yhiM* was regulated by CueR and CpxR, and the CpxAR system was essential for increased Cu resistance in the *ΔyhiM* mutant. Importantly, activation of CpxAR system in the *ΔyhiM* mutant was independent of NlpE, a known activator of this system. YhiM was required for optimal fitness of UPEC in a mouse model of UTI. Our findings demonstrate that YhiM is a critical mediator of Cu homeostasis and links bacterial adaptation to Cu stress with the CpxAR-dependent envelope stress response in UPEC.

**IMPORTANCE:**

UPEC is a common bacterial infection. Bacterial pathogens are exposed to host-derived Cu during infection, including UTI. Here, we describe detection of genes involved in Cu homeostasis in UPEC. A UPEC mutant lacking YhiM, a membrane protein, exhibited dramatic increase in resistance to Cu. Our study demonstrates YhiM as a nexus between Cu stress and the CpxAR-dependent envelope stress response system. Importantly, our findings establish NlpE-independent activation of CpxAR system during Cu stress in UPEC. Collectively, YhiM emerges as a critical mediator of Cu homeostasis in UPEC and highlights the interlinked nature of bacterial adaptation to survival during Cu and envelope stress.

## INTRODUCTION

Urinary tract infection (UTI) is among the most common bacterial infection in humans affecting an estimated 150 million people every year globally (1–3). Uropathogenic *Esherichia coli* (UPEC) is the leading etiological agent of UTI (4, 5). UPEC and other uropathogens cause ascending UTI that begins in the lower urogenital tract proceeding to cause infection and inflammation of the urinary bladder (cystitis) and kidneys (pyelonephritis). UPEC also disseminate to systemic sites and is a major cause of sepsis in the elderly. Multiple virulence and fitness factors have been described in UPEC that allow this pathogen to adhere to the mucosa, colonize the urinary tract, acquire essential nutrients such as iron (Fe), invade and establish intracellular reservoirs, and subvert the host immune response (3, 4, 6–8). Host immune mechanisms take advantage of both essential and toxic micronutrients to hamper pathogen growth during infection (9, 10). Evidence from multiple pathogens and sites of infection, including UPEC and urinary tract, indicate that copper (Cu) is utilized by the host to limit bacterial growth (11–15).

Studies of bacterial response to Cu stress have unraveled the roles of efflux pumps, multicopper oxidases, and buffering ligands in mitigating Cu toxicity (16–18). *E. coli* utilizes CopA pump, CusCFBA efflux system, and CueO multicopper oxidase to decrease total Cu and cuprous Cu content (16, 18). Homeostasis of Cu and Fe in *E. coli* is intricately linked by siderophores (19, 20). The yersiniabactin siderophore system that is found in pathogenic but not commensal *E. coli* strains is involved in protection against Cu toxicity in UPEC (15, 21). Enterobactin production and import facilitated by TonB and its partners are critical for survival of commensal and pathogenic *E. coli* during Cu stress (22). However, our knowledge on import and trafficking of Cu into a bacterial cell remains unclear. To fully harness the anti-microbial activity of host-derived and external Cu for therapeutic applications, it is critical to develop a complete understanding of bacterial Cu homeostasis mechanisms. The objective of the current study was to detect genes involved in Cu homeostasis in *E. coli*. We conducted a forward genetic screen to detect UPEC mutants that exhibit altered sensitivity to Cu, compared to parental strain. Here, we describe the role of YhiM, an inner membrane protein, in modulating Cu homeostasis in UPEC by interacting with the CpxAR envelope stress response system. Our findings elucidate the link between bacterial adaptation to survival under Cu and envelope stress and demonstrates YhiM as a critical protein involved in controlling the activation state of the CpxAR system.

## RESULTS

### Detection of genes that confer Cu sensitivity in UPEC

**Fig 1:**
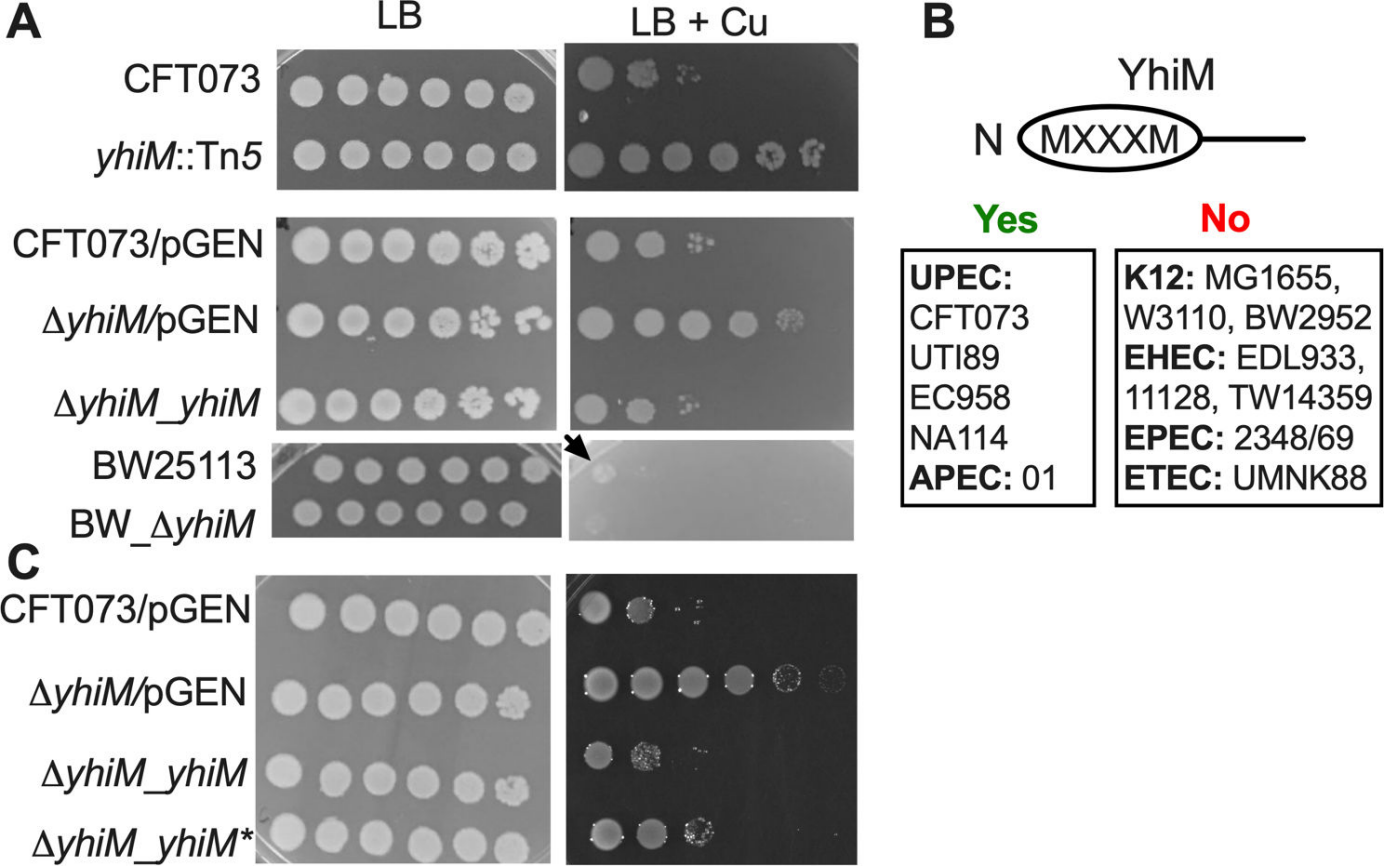
YhiM contributes to Cu sensitivity in uropathogenic *E. coli*. (**A**) Wild-type UPEC (CFT073), Tn*5* insertion mutant (*yhiM::*Tn*5*), empty vector control (CFT073/pGEN), *ΔyhiM* mutant (*ΔyhiM*/pGEN), complemented mutant (*ΔyhiM_yhiM*), laboratory *E. coli* strain BW25113, and *ΔyhiM* mutant (BW_ *ΔyhiM*) were diluted and spot plated on LB or LB with 5-mM CuSO_4_. Arrow indicates growth of BW25113 on Cu-supplemented LB. (**B**) Presence (yes) or absence (no) of a eukaryotic Cu-binding MXXXM motif in the N terminal of YhiM from laboratory and pathogenic *E. coli* strains. (**C**) Growth of empty vector controls (CFT073/pGEN and *ΔyhiM*/pGEN) and complemented mutants (*ΔyhiM_yhiM* and ΔyhiM_yhiM*, encoding a mutant version lacking the XXXM residues) in LB or LB with 5-mM CuSO_4_. A representative image acquired after 24 h of growth from three independent experiments is presented. APEC, avian pathogenic *E. coli*; EHEC, enterohemorrhagic *E. coli*; EPEC, enteropathogenic *E. coli*; ETEC, enterotoxigenic *E. coli*; LB, lysogeny broth.

We took a forward genetic approach by screening a Tn*5* transposon mutant library of 4,608 mutants generated in the UPEC strain CFT073 (Table 1) for growth on Cu-supplemented (6 mM) media (Fig. S1A). We identified 32 mutants (0.7%) that exhibited a higher level of resistance to Cu than the parental strain (Fig. S1B). Transposon insertion sites in these mutants were mapped by rescue cloning approach (Table 2). Only two genes with previously known roles in metal homeostasis were detected in our screen. A Tn*5* insertion in a Cu/silver efflux system gene *cusC* promoted resistance to Cu. In addition, a mutation in *iucC*, a gene involved in the biosynthesis of aerobactin, which is a pathogen-associated siderophore, also led to increased Cu resistance. Twelve of the Cu resistance genes encode proteins that localize in the cytoplasmic or outer membranes, and 5 of them were transmembrane proteins (Table 2). We detected two Cu-resistant mutants in which Tn*5* was inserted in *cpxA,* which encodes the inner membrane sensor kinase/phosphatase that controls the phosphorylation state of CpxR. CpxAR is a two-component regulatory system that orchestrates a response to envelope stress in *E. coli* and other Gram-negative bacteria (23, 24). Furthermore, a mutation in *yhiM*, encoding an inner membrane protein, led to a striking increase in Cu resistance in UPEC (Fig. 1A). In summary, we have determined several genes involved in Cu sensitivity in UPEC. Since the *ΔyhiM* mutant exhibited a remarkable increase in resistance to Cu, we investigated the role of YhiM in Cu homeostasis.

**TABLE 1:** Bacterial strains and plasmids used in this study

Strains or plasmids	Description^*a*^	Reference
CFT073	Wild-type UPEC	(25)
*ΔyhiM*	CFT073 *yhiM::npt*	This study
*ΔcpxA*	CFT073 *cpxA::npt*	This study
*ΔcpxR*	CFT073 *cpxR::npt*	This study
*ΔnlpE*	CFT073 *nlpE::cat*	This study
*ΔyhiMcpxA*	CFT073 *yhiM, cpxA::cat*	This study
*ΔyhiMcpxR*	CFT073 *yhiM, cpxR::cat*	This study
*ΔyhiMnlpE*	CFT073 *yhiM, nlpE::cat*	This study
*ΔcueR*	CFT073 *cueR::npt*	Subash Lab
L-ON	CFT073 *fim* locked ON	(26)
L-OFF	CFT073 *fim* locked OFF	(26)
pGen	Low copy number vector	(27)
pGen_*yhiM*	*yhiM* complementation	This study
pGen_yhiM*	YhiM lacking XXXM	This study
pBAD18	Arabinose-inducible expression vector	(23)
pND18	NlpE overexpression	(23)
pCA24N	IPTG-inducible expression vector^*b*^	(28)
ASKA_*pcpxA*	CpxA expression	(28)
ASKA_*pcpxR*	CpxR expression	(28)
pET28a_*cpxR*	CpxR expression and purification	This study

^*a*^ *npt*, neomycin phosphotransferase; *cat*, chloramphenicol acetyl transferase.

^*b*^ IPTG, isopropyl β-D-1-thiogalactopyranoside.

**TABLE 2:** UPEC genes involved in Cu resistance

Gene	CDS	Annotation	Predicted localization
*nfrA*	c0654	Bacteriophage N4 receptor	Outer membrane
	c4370	Putative membrane protein	Outer membrane
*focD*	c1242	F1C fimbrial usher	Outer membrane
*cusC*	c0658	Outer membrane channel	Outer membrane
*yhiM*	c4289	Hypothetical protein	Inner membrane
*cpxA*	c4863	Sensor histidine kinase	Inner membrane
	c4545	Hypothetical protein	Inner membrane
	c5041	Putative transport sensor protein	Inner membrane
	c4502	Putative antiporter	Inner membrane
*waaH*	c4441	UDP-glucuronate:LPS (HepIII) glycosyltransferase	Inner membrane
*asnA*	c4672	Asparagine synthetase A	Cytosol
*tnaA*	c4631	Tryptophanase	Cytosol
*papI_2*	c5189	P fimbrial operon two regulator	Cytosol
*iucC*	c3625	Aerobactin synthase	Cytosol
*yhgF*	c4184	Putative RNA binding protein	Cytosol
*bglG*	c4646	Transcriptional anti-terminator of cryptic beta-glucosidase operon	Cytosol
*ygfK*	c3456	Hypothetical protein	Cytosol
*ycjG*	c1797	Hypothetical protein	Cytosol
	c5382	Hypothetical protein	Cytosol
	c2508	Hypothetical protein	Cytosol
	c5426	Conserved hypothetical protein	Cytosol
	c5304	Putative conserved protein	Cytosol
	c3171	Putative capsid protein of prophage	Unknown
*ykfF*	c0279	Hypothetical protein	Unknown
	c2748	Hypothetical protein	Unknown
	c0293	Hypothetical protein	Unknown
	c4303	Putative conserved protein	Unknown
Intergenic regions			
c1819 and c1820		Putative transport (c1819), hypothetical protein (c1820)	
c4839 and c4840		Hypothetical protein (c4839), hypothetical protein (c4840)	
c0631 and c0632		Ureidoglycolate dehydrogenase (c0631), FdrA protein (c0632)	
c1208 and c1209		Hypothetical protein (c1208), hypothetical protein (c1209)	
c0307 and c0308		Hypothetical protein (c0307), hemolysin expression modulating protein (c0308)	

### YhiM is involved in Cu homeostasis in UPEC

Involvement of YhiM in Cu homeostasis was further verified with a targeted *ΔyhiM* mutant, generated by Lambda Red recombination (29), and a complemented mutant strain (Table 1). The *ΔyhiM* mutant is more resistant to Cu than its wild-type parental strain, and its Cu sensitivity was reversed to wild-type level by genetic complementation (Fig. 1A). Since *yhiM* is found in the core genome of *E. coli* that is conserved in both commensal and pathogenic strains*,* we tested whether *yhiM* affects growth of a K12 *E. coli* strain BW25113 during Cu stress. Surprisingly, a BW25113 *ΔyhiM* mutant from the KEIO collection (30) did not exhibit enhanced growth in media supplemented with various levels of Cu (Fig. 1; Fig. S2). YhiM is reported to contribute to survival under acid stress in another K12 *E. coli* strain, MG1655 (31). However, the *ΔyhiM* mutant in UPEC strain CFT073 did not exhibit increased sensitivity to acid stress (Fig. S3). Due to these differences in phenotypes, we compared the amino acid sequence of YhiM from commensal and pathogenic *E. coli* strains. Although there was a high level of identity (>90%), an MXXXM motif found in the N terminal of YhiM from UPEC strains was conspicuously absent in commensal and intestinal pathogenic *E. coli* strains (Fig. 1B). The MXXXM motif is highly conserved in the eukaryotic Cu transporter Ctr1, where X is a hydrophobic amino acid and is implicated in Cu transport and in regulating Ctr1 activity (32, 33). To interrogate the role of this motif of YhiM in survival under Cu stress, we constructed a mutant version that lacks the XXXM residues (YhiM*, Table 1). The *ΔyhiM* mutant complemented with the yhiM* mutant construct exhibited increased Cu resistance compared to complementation with the wild-type copy of *yhiM* or the parental wild-type strain (Fig. 1C). However, the yhiM* mutant did not phenocopy Cu resistance of the *ΔyhiM* mutant strain, indicating that the MXXXM motif of YhiM is at least in part responsible for Cu homeostasis in UPEC strain CFT073 (Fig. 1C).

### Mutants lacking YhiM accumulate lower levels of Cu

**Fig 2:**
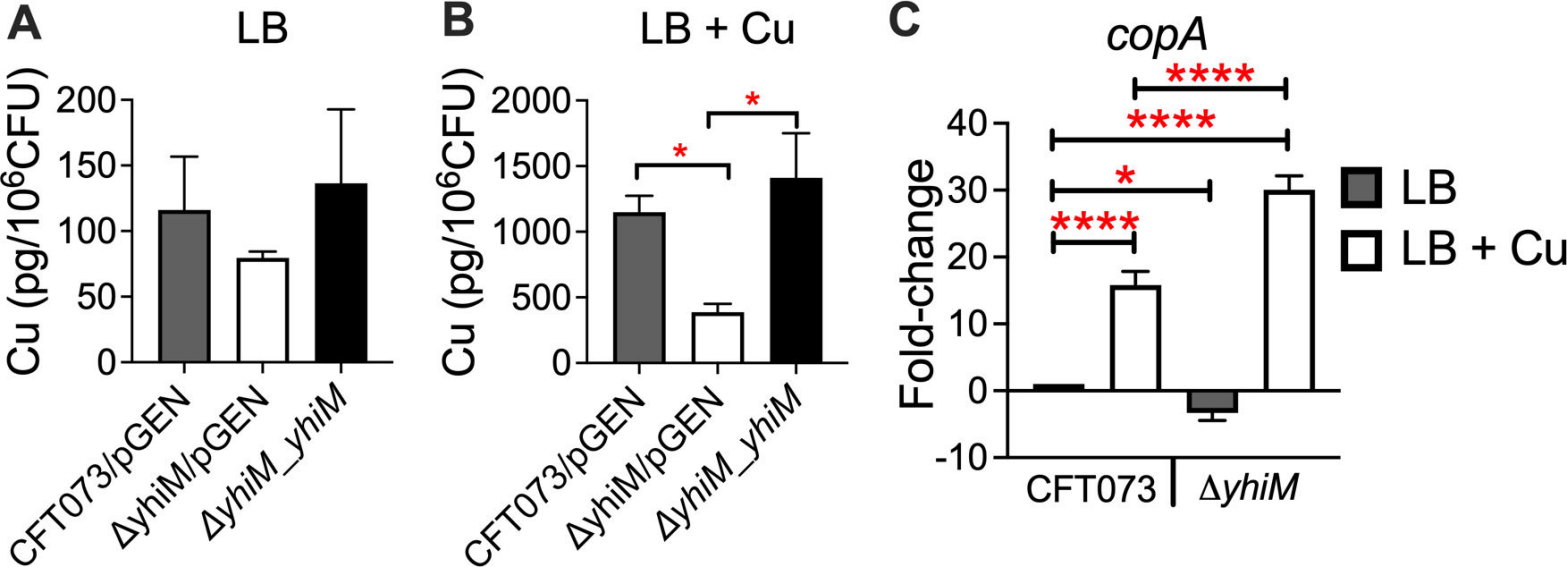
YhiM affects cellular Cu content and *copA* transcript level during Cu stress in UPEC. (A and B) Cu contents of UPEC wild type, *ΔyhiM* mutant, and complemented mutant strains cultured in LB (**A**), or LB with 2-mM CuSO_4_ (**B**) to mid-exponential phase (OD_600_ = 0.5) were determined by ICP-MS and normalized to viable counts. (**C**) Expression of *copA* transcript was quantified in wild type and *ΔyhiM* mutant by real-time PCR. Strains were cultured in LB to mid-logarithmic phase (OD_600_ = 0.5) before exposure to 0.5-mM CuSO_4_ for 20 minutes. Transcript levels were normalized to *gapA,* and relative expression was calculated. Bars depict mean + SEM from three independent experiments. *P < 0.05, ****P < 0.0001. ANOVA with Dunnett’s multiple comparisons test. ANOVA, analysis of variance; pGEN, empty vector; SEM, standard error of the mean.

To interrogate the role of YhiM in Cu homeostasis, we determined the cellular level of key transition metals (Cu, Fe, Mn, and Zn) in wild-type, *ΔyhiM*, and complemented mutant strains by inductively coupled plasma mass spectrometry (ICP-MS). The Cu content of these strains was comparable in the absence of Cu stress (Fig. 2A). The Cu content of *ΔyhiM* was significantly lower than both the wild-type and complemented mutant strains during Cu stress (Fig. 2B). Intracellular levels of iron, manganese, and zinc were comparable in these strains cultured in lysogeny broth (LB) with or without Cu (Fig. S4A through F). Next, we tested whether the expression of Cu efflux genes is increased in the *ΔyhiM* mutant which would lead to lower accumulation of Cu. As expected, Cu induced the expression of the Cu-regulated (CueR) *copA* gene in the wild-type strain (Fig. 2C). However, expression of *copA* was significantly higher in *ΔyhiM* compared to the wild-type strain during Cu stress (Fig. 2C). Also, there were no significant changes in the expression of *cueO*, another CueR-regulated gene, and *cusC*, a CusR-regulated gene in the *ΔyhiM* mutant (Fig. S5A and B).

### Loss of YhiM leads to activation of CpxAR-mediated envelope stress response

**Fig 3:**
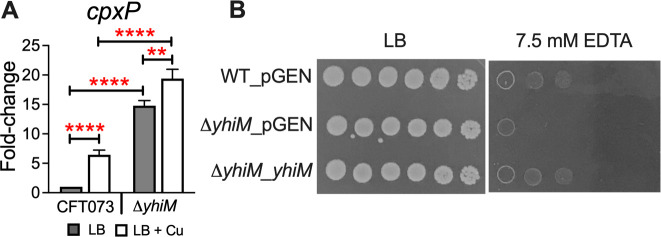
YhiM and Cu-dependent activation of CpxAR envelope stress response system in UPEC. (**A**) Expression of *cpxP* transcript was quantified in wild-type (CFT073) and *ΔyhiM* mutant cultures in LB with or without CuSO_4_ by real-time PCR. Strains were cultured in LB to mid-logarithmic phase (OD_600_ = 0.5) before exposure to 0.5-mM CuSO_4_ for 20 minutes. Transcript levels were normalized to *gapA*, and relative expression was calculated. Bars depict mean + SEM from three independent experiments. **P < 0.01, ****P < 0.0001. ANOVA with Dunnett’s multiple comparisons test. Gray bars denote LB; clear bars indicate LB + 0.5-mM CuSO_4_. (**B**) Envelope stress tolerance of UPEC wild type, *ΔyhiM* mutant, and complemented mutant strains was tested in media containing EDTA. A representative image acquired after 24 h of growth from three independent experiments is presented. pGEN, empty vector.

YhiM is an inner membrane protein (34) consisting of 10 predicted transmembrane domains. Since a Tn mutant in *cpxA* also exhibited increased resistance to Cu (Table 2), we tested whether the increased resistance to Cu in the *ΔyhiM* mutant is linked to the CpxAR system. We determined the expression of *cpxP*, whose transcription is induced by CpxR during envelope stress (35), in the wild-type and *ΔyhiM* strains by quantitative PCR (qPCR). Cu is an activator of the CpxAR envelope stress response system (36, 37), and this was confirmed by our results that revealed a significant upregulation of *cpxP* expression in the wild-type strain during Cu stress (Fig. 3A). Expression of *cpxP* in the *ΔyhiM* mutant was significantly higher than that of the wild-type strain under basal condition, which further increased during Cu stress (Fig. 3A). Since this indicated that the CpxAR system was activated in the *ΔyhiM* mutant, we tested the contribution of YhiM to UPEC survival under envelope stress. The *ΔyhiM* mutant was more sensitive to EDTA than the parental strain, and growth was rescued by genetic complementation (Fig. 3B).

### Transcription of *yhiM* is positively regulated by the CpxAR system and negatively regulated by CueR

**Fig 4:**
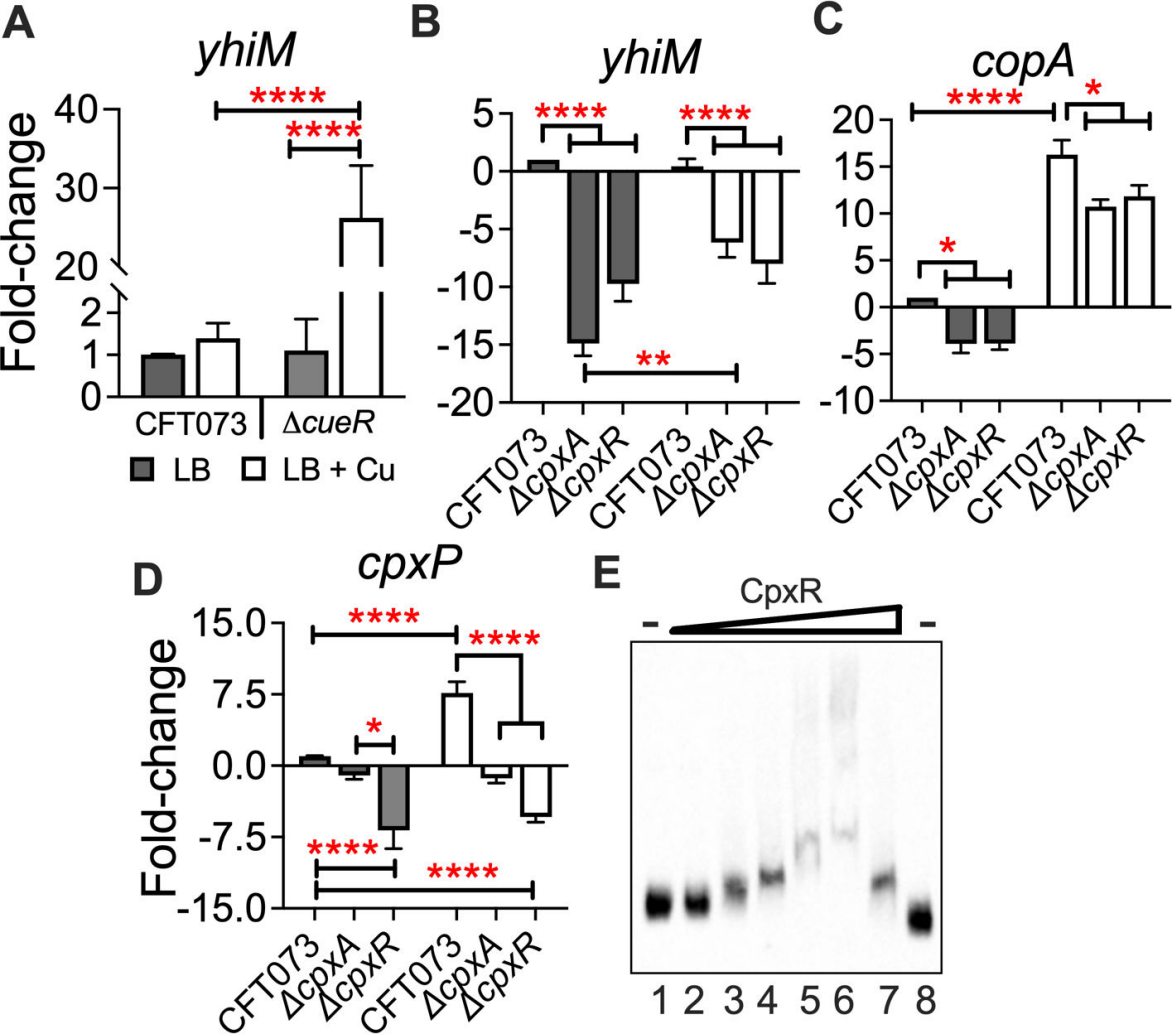
CueR and CpxAR regulate the expression of *yhiM* in a Cu-dependent manner. Wild-type and mutant strains were cultured in LB to mid-logarithmic phase (OD_600_ = 0.5) before exposure to Cu for 20 minutes. Levels of *yhiM* (A and B), *copA* (**C**), and *cpxP* (**D**) transcripts were quantified by real-time PCR. Transcript levels were normalized to *gapA*, and relative expression was calculated. Bars depict mean + SEM from three independent experiments. *P < 0.05, **P < 0.01, ****P < 0.0001, ANOVA with Dunnett’s multiple comparisons test. Gray bars denote LB; clear bars indicate LB + 0.5-mM CuSO_4_. (**E**) Biotinylated probe of *yhiM* (40 ng/µL, 0 to −400 bp) was incubated in the presence of 0 mg (lanes 1 and 8), and increasing concentrations (1–5 mg, lanes 2–7) of phosphorylated CpxR. Unlabeled probe (lane 7) and BSA (5 µg, lane 8) were used as controls. Samples were electrophoresed, transferred to nylon membranes, and cross-linked, and chemiluminescence was detected. A representative image from three independent experiments is presented. BSA, bovine serum albumin.

Since YhiM affects UPEC survival during both Cu and envelope stress, we determined the extent to which expression of *yhiM* is regulated by CueR and envelope stress-responsive (CpxR) transcriptional regulators. To evaluate the role of the CpxAR system in Cu stress and its connection to YhiM in UPEC, we generated genetically defined *ΔcpxA* and *ΔcpxR* mutants in UPEC strain CFT073 (Table 1). Abundance of *yhiM* transcripts was determined and compared between wild-type, *ΔcueR*, *ΔcpxA,* and *ΔcpxR* mutants cultured in LB with or without Cu. Under basal conditions, CueR did not affect *yhiM* transcript levels in UPEC (Fig. 4A). Cu stress induced a marked increase in the expression of *yhiM* in the *ΔcueR* mutant compared to the wild-type strain and the *ΔcueR* mutant cultured in LB (Fig. 4A). However, loss of CpxA and CpxR resulted in decreased abundance (~10-fold) of *yhiM* transcript in control and Cu-stressed UPEC (Fig. 4B). The *cpxA* mutant had higher level of *yhiM* transcript during Cu stress compared to LB alone (Fig. 4B). There was no Cu-dependent change in *yhiM* transcript level in the *ΔcpxR* mutant (Fig. 4B). Since CpxR is known to regulate transcription of *copA* in commensal *E. coli (37*), we tested *copA* expression in UPEC *ΔcpxA* and *ΔcpxR* mutants. Lower levels of *copA* transcript were detected in the absence of CpxAR relative to the wild-type strain in LB (Fig. 4C). Although *copA* was expressed at a higher level during Cu stress in these mutants, it remained significantly lower than the wild-type strain (Fig. 4C).

We investigated the activation state of the Cpx pathway in the *ΔcpxA* and *ΔcpxR* mutants by determining the levels of *cpxP* transcript. The abundance of *cpxP* transcript was significantly lower in the *ΔcpxR* mutant compared to the wild-type control and the *ΔcpxA* mutant, consistent with its role as a transcriptional activator of *cpxP* in the absence and presence of Cu stress (Fig. 4D). In contrast, levels of the *cpxP* transcript were significantly lower in the *ΔcpxA* mutant compared to the wild-type control during Cu stress (Fig. 4D). Electrophoretic mobility shift assay (EMSA) was conducted to probe whether CpxR is directly involved in regulating the expression of *yhiM* (Fig. 4E). Purified CpxR (Fig. S6) was phosphorylated and incubated with the biotin-labeled promoter region of *yhiM* and controls (unlabeled probe and albumin). Binding of CpxR with the *yhiM* promoter region was detected by the decreased mobility of the labeled probe with increasing concentrations of CpxR (Fig. 4E). The presence of BSA did not affect the mobility of the labeled probe (Fig. 4E). Collectively, these results reveal that CueR negatively regulates *yhiM* expression in contrast to CpxAR, which serves as a positive regulator of *yhiM* expression.

### CpxA and CpxR contribute to UPEC survival during Cu stress

**Fig 5:**
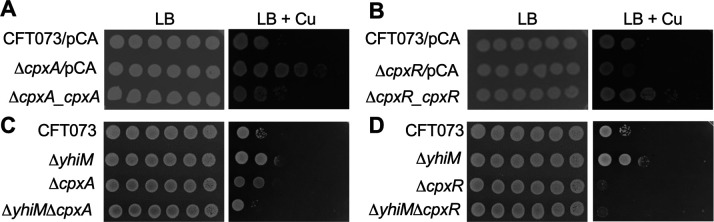
CpxA, CpxR, and YhiM are involved in growth during Cu stress. Wild-type, mutant, and complemented mutant strains, indicated in panels A–D, were adjusted to equal turbidity (OD_600_ = 1), diluted and spot plated on LB agar with or without 5-mM CuSO_4_. Expression of *cpx* genes was induced with IPTG. A representative image acquired after 24 h of growth from three independent experiments is presented here. pCA, empty vector (pCA24N).

Cu induces envelope stress leading to the activation of the CpxAR system in UPEC (Fig. 3A), which is consistent with previous reports on laboratory strains of *E. coli* (37, 38). First, we confirmed the known increase in sensitivity to EDTA in *ΔcpxA* and *ΔcpxR* mutants in UPEC (Fig. S5). We assessed the sensitivity of UPEC mutants lacking CpxA and CpxR to Cu stress. The *ΔcpxA* mutant was more resistant to Cu, whereas the *ΔcpxR* mutant was more sensitive to Cu than the wild-type strain (Fig. 5A and B). Opposing phenotypes of these mutants are in line with previous reports on increased transcriptional activity of CpxR in mutants lacking CpxA (24) and are supported by our findings (Fig. 4D). These Cu-responsive phenotypes could be restored to wild-type levels by complementation with *cpxA* and *cpxR* genes expressed from an IPTG-inducible promoter (Fig. 5A and B). To probe whether increased Cu resistance of the *ΔyhiM* mutant is CpxAR dependent, *ΔyhiMΔcpxA* and *ΔyhiMΔcpxR* double mutants were constructed, and their growth was evaluated on LB and Cu-supplemented agar. The *ΔyhiMΔcpxA* mutant was more sensitive to Cu than single mutants lacking either YhiM or CpxA and also than the wild-type strain (Fig. 5C and D). The *ΔyhiMΔcpxR* mutant was more sensitive to Cu than wild-type strain, and it phenocopied the *ΔcpxR* mutant (Fig. 5C and D). Cumulatively, our results reveal an important connection between YhiM and the activation status of the CpxAR system in promoting UPEC growth during Cu stress.

### NlpE-independent activation of the CpxAR system in UPEC

**Fig 6:**
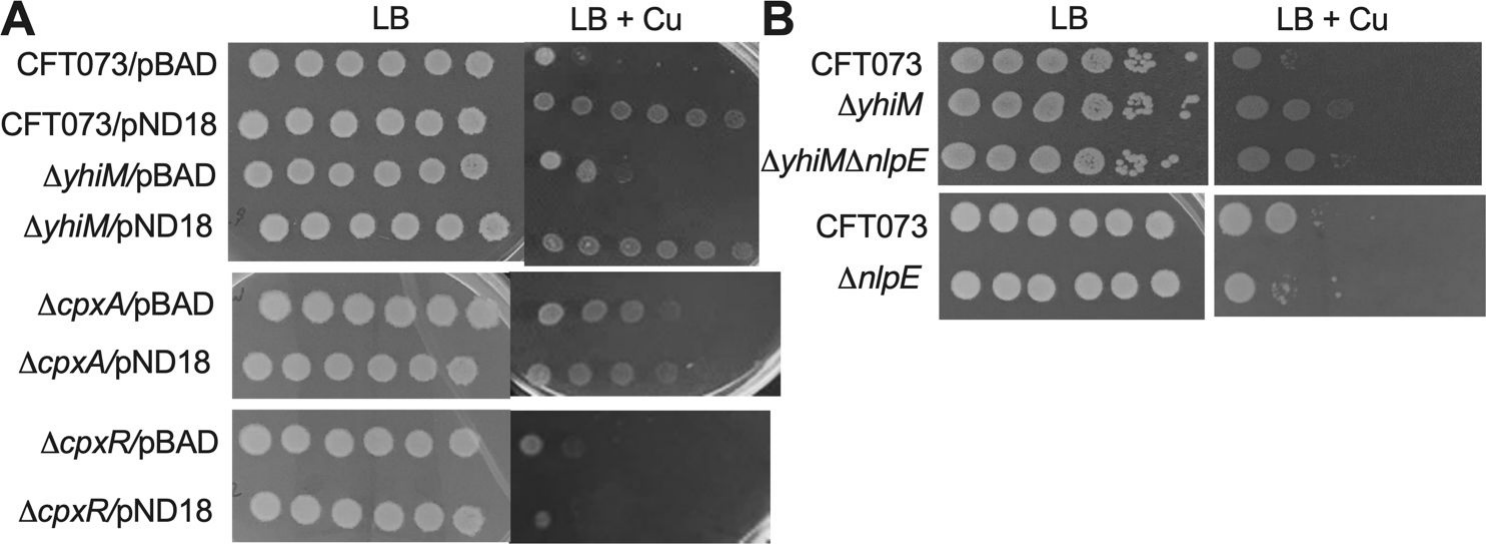
NlpE augments UPEC survival during Cu stress in a CpxAR-dependent, and YhiM-independent manner. (**A**) Wild-type and indicated mutant strains harboring empty (pBAD) or *nlpE* overexpression vectors (pND18) were diluted and spot plated on LB agar with or without 5-mM CuSO_4_. Expression of *nlpE* was induced with arabinose. (**B**) Sensitivity of wild-type, *ΔyhiM*, *ΔyhiMΔnlpE*, and *ΔnlpE* mutant strains to Cu stress was evaluated as described here for panel A. A representative image acquired after 24 h of growth from three independent experiments is presented here.

**Fig 7:**
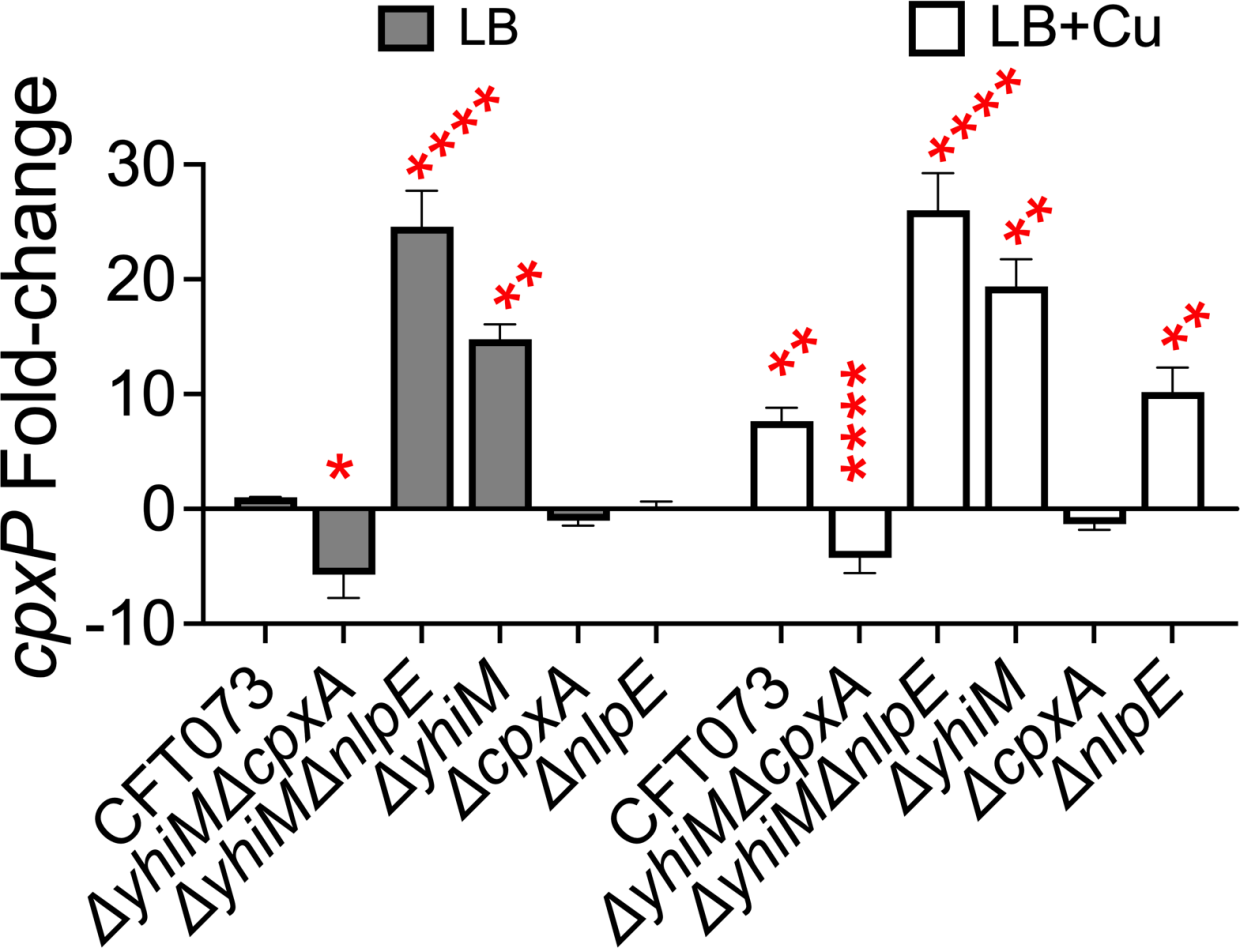
CpxA but not NlpE is involved in *cpxP* expression in the absence of YhiM. (A and B) Wild-type and indicated mutant strains were cultured in LB to mid-logarithmic phase (OD_600_ = 0.5) before exposure to Cu for 20 minutes. Levels of *cpxP* transcript were quantified by real-time PCR. Transcript levels were normalized to *gapA,* and relative expression was calculated. Bars depict mean + SEM from three independent experiments. *P < 0.05, ****P < 0.0001, ANOVA with Dunnett’s multiple comparisons test. Gray bars denote LB; clear bars denote LB + 0.5-mM CuSO_4_.

Next, we investigated the role of NlpE in activating the CpxAR system in the *ΔyhiM* mutant during Cu stress due to the involvement of NlpE in activating the CpxAR system and its interaction with Cu. An *nlpE* overexpression construct (pND18) and vector control (pBAD) were introduced into CFT073 wild-type, *ΔyhiM*, *ΔcpxA,* and *ΔcpxR* strains, and Cu sensitivity assays were performed. Overexpression of *nlpE* in the wild-type strain resulted in increased resistance to Cu stress (Fig. 6A). Increasing the level of NlpE in the *ΔyhiM* mutant did not increase its resistance to Cu above that of the wild-type strain (Fig. 6A). Additionally, the NlpE-dependent increase in Cu resistance was abrogated in the *ΔcpxA* and *ΔcpxR* mutants (Fig. 6A). To further probe the connection between YhiM and NlpE, a *ΔyhiMΔnlpE* double mutant was constructed. The *ΔyhiMΔnlpE* mutant was more resistant to Cu than the wild type, and comparable to that of the *ΔyhiM* mutant (Fig. 6B). However, the *ΔnlpE* mutant was more sensitive to Cu than the parental strain (Fig. 6). Since abundance of the *cpxP* transcript was higher in the *ΔyhiM* mutant (Fig. 3A), we tested the role of CpxA and NlpE in mediating increased *cpxP* levels. Lower levels of *cpxP* were observed in the *ΔyhiMΔcpxA* mutant under basal and Cu stress conditions, compared to the wild-type strain (Fig. 7). In contrast, the *cpxP* transcript level was more elevated in the *ΔyhiMΔnlpE* and *ΔyhiM* mutants than the wild-type strain, in both basal and Cu stress conditions (Fig. 7). There was no change in the *cpxP* transcript levels in the *ΔcpxA* mutant under basal and Cu stress conditions (Fig. 7). Interestingly, the *cpxP* transcript levels were elevated in the *ΔnlpE* mutant only during Cu stress (Fig. 7). Taken together, our results indicate that the activation the CpxAR system in the *ΔyhiM* mutant is independent of NlpE.

### Role of YhiM in UPEC fitness during UTI in a mouse model

**Fig 8:**
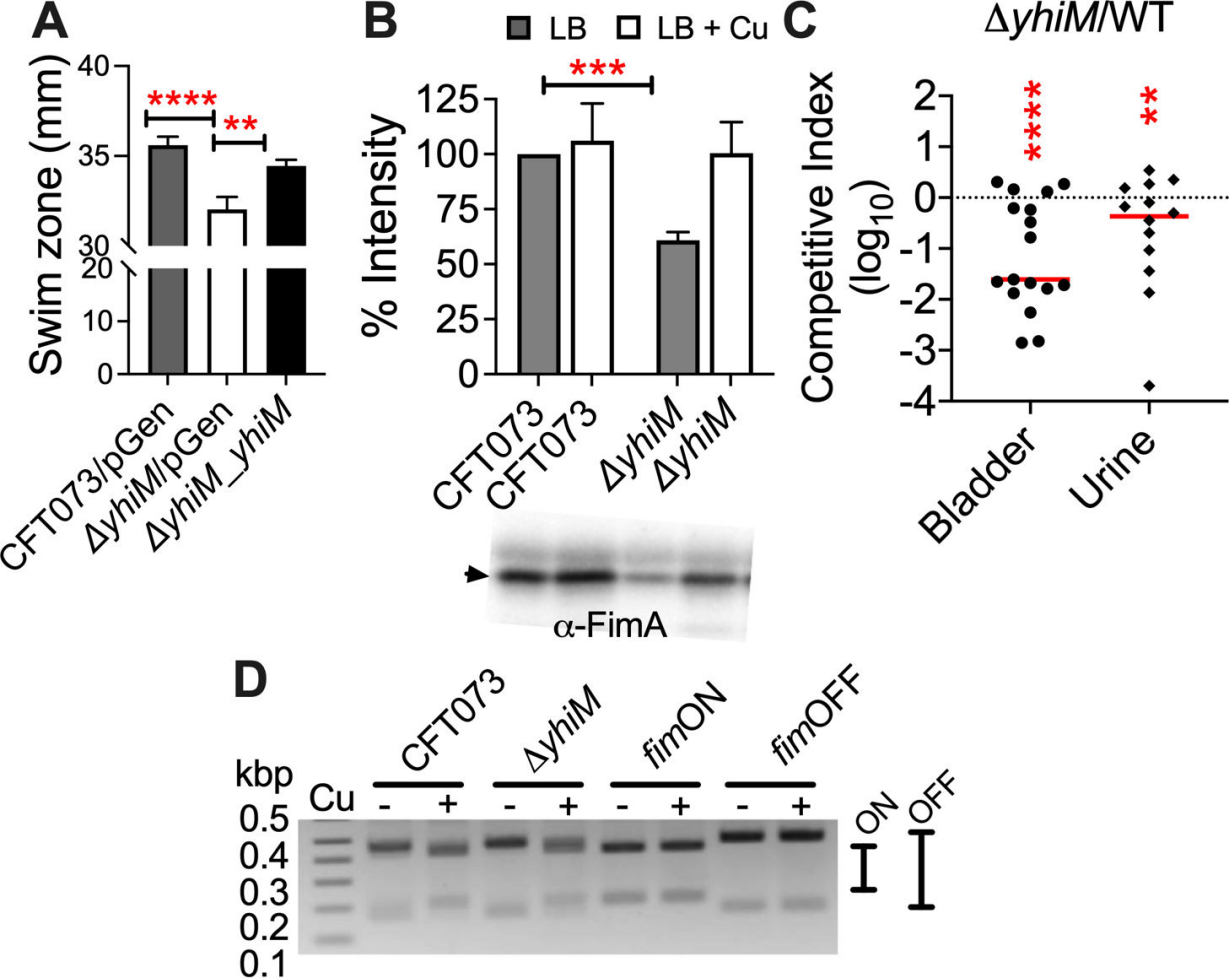
YhiM is involved in UPEC fitness during UTI in a mouse model. (**A**) Swimming motility of wild-type (CFT073), *ΔyhiM* mutant, and complemented mutant was determined in soft agar. (**B**) Levels of FimA subunit of type 1 pili were determined in the wild-type and *ΔyhiM* mutant strain cultured in LB with or without 0.5-mM CuSO_4_ by immunoblotting. Images from three independent experiments were used to quantify signal intensity. A representative blot is depicted here. Arrow indicates 18 kDa. **P < 0.01, ***P < 0.001. ANOVA with Dunnett’s multiple comparisons test. (**C**) Female mice were inoculated with a 1:1 mixture of wild-type strain and *ΔyhiM* mutant in the urinary bladder. Competitive indices were calculated as the ratio of mutant to wild-type strain *in vivo*, normalized to their ratio in the inoculum. Each symbol corresponds to results from a mouse, and bars indicate the median. Dotted line, no loss of fitness in the mutant relative to wild-type strain (competitive index of 1). (**D**) Invertible element PCR assay in the presence or absence of Cu depicting the *fim* promoter in the on or off orientation. **P < 0.01, ****P < 0.0001. Wilcoxon signed-rank test. pGEN, empty vector.

YhiM is involved in swimming motility of UPEC, since the *ΔyhiM* mutant exhibited a modest but statistically significant decrease in motility relative to the parental strain, which was restored in the complemented mutant strain (Fig. 8A). CpxR activation is known to decrease the expression of adhesive appendages including type one and other pili in UPEC (39, 40). Since we observed activation of the CpxAR system in the *ΔyhiM* mutant, we tested the production of type one fimbria, an important colonization factor which mediates UPEC adhesion to epithelial cells during the infection (41). Production of a type 1 fimbrial subunit, FimA, was determined by Western blotting in the presence and absence of Cu stress in UPEC. FimA production was noticeably decreased in the *ΔyhiM* mutant in uninduced conditions, and Cu stress restored the FimA levels to that of the parental strain (Fig. 8B; Fig. S8). The *fim* promoter was predominantly present in the off orientation in the wild-type and *ΔyhiM* mutant strains (Fig. 8D). However, the wild-type strain contained a faint band corresponding to the on orientation, and this was not found in the *ΔyhiM* mutant (Fig. 8D). Induction of Cu stress led to the *fim* promoter switching to the on orientation, regardless of the presence of *yhiM* (Fig. 8D). Next, we investigated whether YhiM is involved in pathogen fitness in the murine urinary tract by performing co-infection experiments. Female CBA/J mice were infected with equal numbers of wild-type UPEC CFT073 and *ΔyhiM* (5 × 10^7^ CFU each). Bacterial burden in urine and tissue samples was determined at 48 hpi and used to calculate competitive indices (Fig. 8C; Fig. S9). The *ΔyhiM* mutant exhibited a significant decrease in fitness relative to the parental strain in urine and in the urinary bladder (Fig. 8C). There was a trend toward reduced fitness of the mutant in kidneys and systemic sites (spleen and liver), but this difference was not statistically significant (Fig. S9). In summary, YhiM is involved in swimming motility, type 1 fimbria production, and fitness of UPEC in a mouse model of UTI.

## DISCUSSION

**Fig 9:**
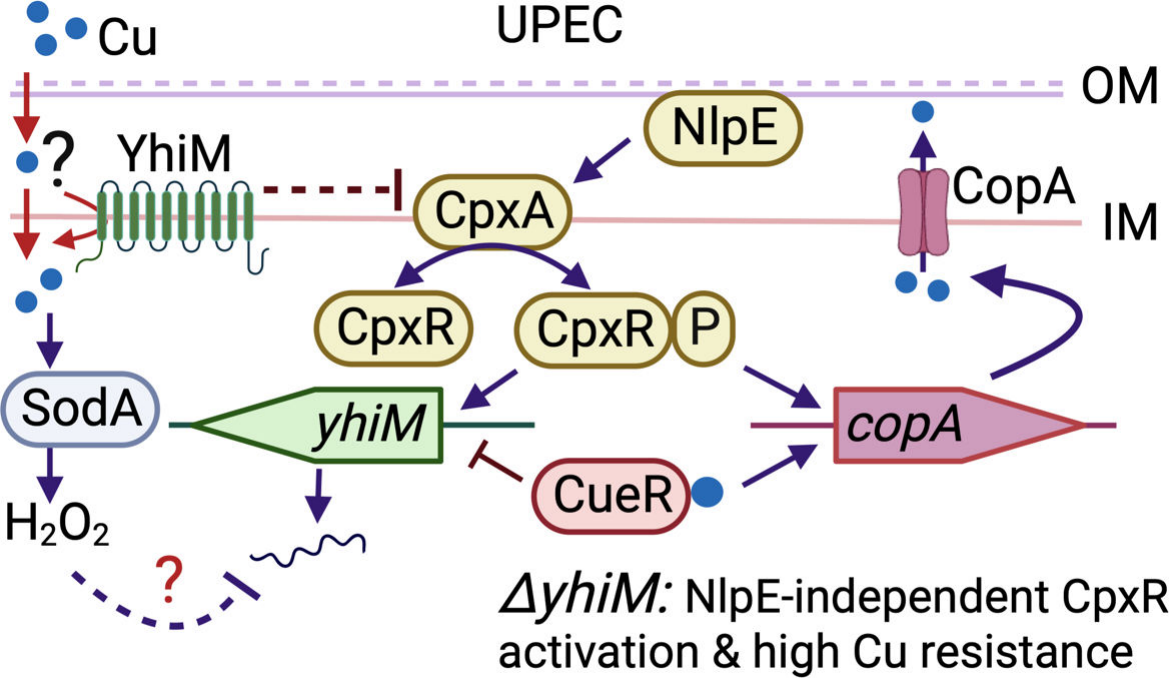
YhiM connects Cu stress with the CpxAR system in UPEC. Excess intracellular Cu activates CueR to increase the transcription of *copA,* which encodes a P-type ATPase involved in Cu efflux, and to inhibit transcription of *yhiM*. YhiM inhibits activation of the CpxAR system in UPEC. Cu stress also activates superoxide and hydrogen peroxide response systems that could also affect the expression of *yhiM*. The *ΔyhiM* mutant has increased Cu resistance due to constitutive induction of the CpxAR system in an NlpE-independent mechanism. Collectively, these responses work to minimize YhiM levels during Cu stress to achieve optimal activation of the CpxAR system and mitigate the toxic effects of Cu. IM, inner membrane; OM, outer membrane; UPEC, uropathogenic *E. coli*.

Cu is increasingly implicated as an effector of nutritional immunity, an arm of the innate immune response, for protection against bacterial pathogens (7, 9, 11). We and others have demonstrated that UPEC and other uropathogens are exposed to Cu in the inflamed urinary tract (12–15, 21). UPEC utilizes yersiniabactin, a siderophore, to not only acquire iron but also to mitigate Cu toxicity (15, 21). A UPEC mutant lacking the CusCFBA Cu efflux system exhibits decreased fitness in a murine model of UTI (12). Cu efflux (CopA and CusCFBA) and detoxification (CueO) mechanisms are well characterized in *E. coli* (16, 20). However, the identity of transporters involved in import and trafficking of Cu across the Gram-negative bacterial cell envelope remains unclear. Here, we utilized a forward genetic screen to detect genes that are potentially involved in Cu import. We hypothesized that transposon mutants with inactivated Cu import genes will exhibit increased resistance to Cu compared to the parental strain. Our screen has revealed multiple unique candidate genes that are involved in Cu homeostasis in *E. coli*. In this report, we focus on the role of YhiM, an inner membrane protein, and its interaction with the CpxAR system in modulating Cu homeostasis in UPEC (Fig. 9).

YhiM is an inner membrane protein that contains a domain of unknown function (DUF2776). Production and localization of YhiM have been experimentally verified in *E. coli* (34). Our results demonstrate a role for this protein in mitigating Cu toxicity in UPEC but not in commensal *E. coli*. Previous reports have indicated a role for YhiM in survival under acidic pH in commensal *E. coli* (31). We were interested in this connection because Cu toxicity to UPEC is accentuated by acidic pH (42). Our experiments did not reveal a role for YhiM in survival under acidic conditions in UPEC, suggesting that the function of YhiM is based on the genetic background of the *E. coli* strain. Due to extensive genetic heterogeneity, the pangenome of *E. coli* isolates contains ~55,000 genes (43–45). In contrast, the core genome of *E. coli* that is conserved across sequenced isolates is composed of ~2,200 genes (43–45). Our findings reveal that there are minor variations in genes that constitute the core genome of *E. coli* with significant functional consequences. Our findings also implicate a role for the MXXXM motif of YhiM during Cu stress in UPEC strain CFT073. Although YhiM is highly conserved among commensal and pathogenic *E. coli* strains, it plays strikingly different roles in resistance to Cu and acid stress. Our observation highlights the importance of considering the potential roles of conserved genes in phenotypes that are linked to pathogenesis and interaction with the host.

Exposure to Cu activates the transcription of Cu efflux and detoxification genes (36, 37) and is confirmed by our results (Fig. 9). Our findings demonstrate that the *ΔyhiM* mutant accumulates less cell-associated Cu compared to the wild-type strain by activating the transcription of *copA*. However, the *ΔyhiM* mutant contains a higher level of Cu during Cu stress compared to LB, indicating that Cu import is not abrogated in the absence of YhiM. Additional regulatory mechanisms that coordinate bacterial survival under envelope, superoxide, and hydrogen peroxide stress are also activated during Cu stress in *E. coli* (36, 37, 46). Cu-induced superoxide stress leads to an increased superoxide dismutase activity of SodA and SodC in UPEC (46). Dismutation of superoxide yields hydrogen peroxide, a highly reactive molecule that needs to be neutralized by catalases and peroxidases. OxyR is a central player in responding to hydrogen peroxide (47), and Cu stress triggers the transcription of genes in the OxyR regulon in commensal and pathogenic *E. coli* (46, 48). OxyS is a small non-coding RNA which acts as a post-transcriptional regulator that orchestrates cellular resistance to hydrogen peroxide (49). Transcription of *oxyS* is induced by OxyR, and OxyS decreases the expression of *yhiM* in *E. coli* (49). Considering our finding on increased Cu resistance in the *ΔyhiM* mutant, suppression of *yhiM* expression by OxyS during Cu stress emerges as a plausible mechanism that promotes survival of *E. coli*.

Understanding the cellular response to stressors affecting the bacterial cell envelope has significant ramifications for deciphering events during host-pathogen interaction and for developing next-generation anti-microbial agents. Signaling via the CpxAR system, including the identity of envelope proteins critical for recognizing and transducing signals, is well characterized in *E. coli* (50, 51). The CpxAR system is involved in the virulence of UPEC strain UTI89 during pathogenesis of cystitis and is required for optimal adhesion and invasion of UPEC to a human urothelial cell line (52). CpxR is a transcriptional repressor of hemolysin in UPEC, and a *ΔcpxR* mutant that overproduces hemolysin is attenuated during acute and chronic cystitis in mice (53). However, published reports paint a complex portrait of the relationship between CpxAR and UPEC virulence due to strain-specific variation. Mutants that lack *cpxA* (high levels of CpxR activity) and *cpxR* (no CpxR activity) in UPEC stain CFT073 are both attenuated in a mouse model of UTI (39). Our findings reveal that the *ΔyhiM* mutant in UPEC strain CFT073 has elevated CpxR activity, and decreased fitness in the murine urinary bladder. The contribution of CpxR activation to the fitness defect of the *ΔyhiM* mutant should be evaluated in future studies with *ΔyhiMΔcpxA* and *ΔyhiMΔcpxR* mutants to delineate Cpx-dependent and Cpx-independent effects on UPEC fitness in the mouse model of UTI. Here, we demonstrate a critical role for the CpxAR system in regulating UPEC survival during Cu stress (Fig. 9). Our observations are in accordance with previous reports on the role of the CpxAR system during Cu stress in commensal *E. coli* (37, 38, 54). Collectively, the CpxAR system affects many facets of UPEC adaptation to survival during exposure to host-associated stressors.

Transcriptomic analysis has revealed that expression of *yhiM* is regulated by the CpxAR system in UPEC (39) and is complemented by our real-time PCR results. However, there is no detectable CpxR-binding consensus sequence in the promoter region of *yhiM* explaining why this gene was not detected earlier in genome scans in *E. coli* (54, 55). Here, we demonstrate that CpxR indeed binds to the *yhiM* promoter, and specificity of this interaction was established by outcompeting with an unlabeled probe. The *ΔcpxA* mutant did not exhibit increased activation of the CpxAR system under the growth conditions used in our studies. Consistent with the role of CpxR as an activator, *yhiM* transcript levels are lower in the *ΔcpxA* and *ΔcpxR* mutants, relative to the parental strain. Loss of Cu resistance in the *ΔyhiMΔcpxA* and *ΔyhiMΔcpxR* double mutants provides strong genetic evidence that demonstrates that CpxAR system plays a critical role in Cu resistance of the *ΔyhiM* mutant. Our real-time PCR assays reveal that optimal expression of *yhiM* requires the CpxAR system. However, there is lower *yhiM* expression in the *ΔcpxA* mutant compared to the *ΔcpxR* mutant. The strikingly different Cu resistance phenotypes of the *ΔcpxA* and *ΔcpxR* mutants is also supported by genetic complementation where *cpxR* but not *cpxA* leads to increased Cu resistance. Our studies also raise the possibility of additional effectors and mechanisms that could phosphorylate CpxR in the *ΔcpxA* mutant that could lead to increased Cu resistance. Furthermore, the absence of CpxR leads to most significant Cu sensitivity among the mutants presented in this report. Since loss of CpxR does not completely reinstate *yhiM* transcript levels, our results suggest that there are additional regulators likely involved in controlling the expression of *yhiM*.

NlpE is an outer membrane lipoprotein whose overexpression results in the activation of the CpxAR system and transcription of genes in the CpxR regulon (56). An *E. coli* mutant lacking *nlpE* (*cutF*) was more sensitive to Cu than the wild-type strain (57) and is consistent with our findings. May et al. have reported that the N-terminal domain of NlpE that is mislocalized in the inner membrane activates the CpxAR system during Cu stress (38). We probed whether activation of the CpxAR system in the *ΔyhiM* mutant is mediated by NlpE during Cu stress. Our results reveal that the *ΔyhiMΔnlpE* double mutant is more resistant to Cu and has elevated levels of *cpxP* transcript than the wild-type strain, indicating activation of the CpxAR system in the double mutant. Taken in light of the previously known role of NlpE as a sensor for lipoprotein trafficking (38), our findings also suggest that YhiM is less likely to be involved in lipoprotein biogenesis and trafficking. These data point to a model where loss of YhiM activates the CpxAR system in an NlpE-independent manner and raise questions on direct versus indirect interaction between YhiM and CpxA. Identification of YhiM here as a modulator of the CpxAR signal transduction system suggests the potential presence of additional intermediaries involved in controlling the activation state of the CpxAR system. In summary, YhiM emerges as a connecting link between Cu and envelope stress responses in UPEC strain CFT073 (Fig. 9).

A limitation of the current study is the lack of a genome-saturating mutant library. Since our transposon library has 4,608 mutants, it is plausible that there are additional genes involved in modulating Cu homeostasis in *E. coli* that were not detected in our screen. Additionally, UPEC strains are genetically heterogenous, raising the possibility of the existence of lineage-specific Cu resistance genes and regulation of the CpxAR system. Here, we report the presence of the MXXXM motif in YhiM from UPEC strains but not in enteric *E. coli* (both commensal and pathogenic) strains. Further studies in other prototypical UPEC strains such as UTI89 and EC958 are needed to determine the degree of conservation of the role of YhiM in combating Cu stress and activating the CpxAR system. Future studies utilizing a transposonomic approach, previously applied in UPEC to detect genes involved in bacteremia and systemic colonization (58) in prototypical UPEC strains could overcome the limitation of a lack of genome saturation.

Our findings indicate a clear role for YhiM in affecting Cu homeostasis in UPEC and underscores the interconnected nature of bacterial adaptation to Cu and envelope stress. Ongoing studies in our group are addressing the mechanism(s) by which YhiM impacts UPEC survival under Cu stress, including the direct role of YhiM in interacting with and importing Cu. In summary, YhiM is a critical modulator of Cu homeostasis that connects Cu stress with CpxAR-based envelope stress response in UPEC strain CFT073.

## MATERIALS AND METHODS

### Bacterial strains and mutant construction

Clinical UPEC strain CFT073 was isolated from the urine and blood of a patient (25). Targeted mutations were introduced into UPEC strain CFT073 by lambda red recombineering (29). Successful introduction of mutations was verified by PCR with primers that bind to the antibiotic resistance cassette and the gene of interest. Strains and oligonucleotide primers are listed in Table 1 and Table S1, respectively.

### Culture conditions and reagents

Strains were inoculated in LB broth or agar (tryptone, 10 g/L; yeast extract, 5 g/L; NaCl, 5 g/L; and agar, 15 g/L). Cultures were incubated at 37°C, with shaking at 200 RPM, unless noted otherwise. An optical density of OD_600_ = 0.5 was considered as the mid-logarithmic phase. Chemicals were purchased from Sigma, and exceptions are indicated.

### Genetic complementation

Complementation plasmids were constructed by cloning the full-length *yhiM* gene along with a 500-bp region upstream of their start codon into a low-copy number plasmid, pGEN-MCS (27). Amplicons were generated using primers listed in Table S1 and were cloned into pCR4 Topo (Invitrogen). The *yhiM* gene and its promoter was released by digestion with EcoRI, cloned into EcoRI site of pGEN-MCS, and verified by PCR and Sanger sequencing. A mutant version of *yhiM* that does not encode the XXXM residues of the MXXXM motif was also generated and verified by Sanger sequencing. Plasmids used in this study are listed in Table 1.

### Generation of a transposon mutant library

Random insertional mutations were introduced in UPEC strain CFT073 with Ez-Tn*5* transposomes (Epicentre/Lucigen), as we have previously described (58). Briefly, transposome complexes were electroporated into competent cells, and transformants were selected on LB agar plates containing 25-µg/mL kanamycin. Four thousand six hundred eight individual colonies were archived in 96-well plates.

### Screen for Cu-resistant mutants

Transposon mutants and controls (wild-type and CFT073 *ΔcopA* strains) were cultured overnight and screened for Cu resistance on LB agar with 6-mM CuSO_4_ by spot plating 3 µL in 8 × 12 format. This concentration of Cu was selected since it inhibits the growth of the wild-type strain and is in line with previous reports on *in vitro* assessment of Cu resistance in rich media (22, 38, 46, 59, 60). Secondary screen was conducted by spot plating serial dilutions of mutants detected in the primary screen, to verify the Cu resistance phenotype. Assays were independently repeated three times.

### Detection of transposon insertion sites

Genomic DNA extracted from Cu-resistant mutants was digested with *Pvu*II, ligated with T4 DNA ligase, and transformed into *E. coli* strain DH5 lambda *pir* competent cells. Plasmids were prepped from kanamycin-resistant transformants, and Sanger sequencing with primers listed in Table S1 was used to identify transposon insertion sites.

### Cu and EDTA sensitivity assays

Overnight cultures of bacterial cells were diluted, and 10-fold dilutions were spot plated on LB agar containing up to 6 mM CuSO_4_. As indicated in figure legends, arabinose, ampicillin, or IPTG supplemented media were used. Images were acquired after overnight incubation at 37°C. Sensitivity to EDTA was also determined essentially as described here for Cu but with up to 10-mM EDTA.

### qPCR

Overnight cultures were subcultured in LB broth and grown to mid-logarithmic phase (OD_600_ = 0.5) before exposure to 0.5-mM CuSO_4_ for 20 minutes. RNAprotect (Qiagen) was added to stabilize the transcripts, and cells were harvested by centrifugation. RNA was extracted (RNeasy, Qiagen) and treated with DNase (Ambion) to eliminate DNA contamination. cDNA synthesized with Superscript III reverse transcriptase (Invitrogen) was used in SYBR green-based qPCR (Thermo Scientific) performed with oligonucleotide primers (Table S1) in a CFX Real-Time system (Bio-Rad). Transcript levels were normalized to *gapA*, and relative expression was determined using untreated controls of each strain as the calibrator.

### FimA Western Blot

FimA levels were determined as described earlier (61). Strains were cultured in LB with or without 0.5-mM CuSO_4_ static for 24 h. OD_600_ of overnight cultures were adjusted to 1.0, acidified with water (pH 1.8), and denatured. An equal amount of protein was separated on 15% SDS-PAGE and transferred to 0.45-μm PVDF membrane (Millipore). Blots were blocked with 5% skim milk, probed with FimA antibody (1:10,000 dilution), detected with anti-rabbit IgG secondary antibody (1:10,000 dilution, Invitrogen), and visualized with ECL-Prime reagents (Amersham). Images were acquired in a Chemidoc system (Biorad). Duplicate gels were stained with Commassie blue to serve as loading controls.

### Invertible element PCR

Bacterial strains were subcultured in LB at 37°C without shaking for 2 h. CuSO_4_ at 0.5 mM or sterile water was added, and incubation was continued overnight at 37°C. OD_600_ was adjusted to 1, and cells were harvested and resuspended in deionized water. The supernatant from boil preps was used as the PCR template. The 601-bp invertible element was amplified (primers listed in Table S1), digested with *SnaBI*, and separated on 2% agarose gel to determine the orientation of invertible element (on or off orientation).

### ICP-MS

Bacteria cultured in LB or LB containing 2-mM CuSO_4_ to OD_600_ of 0.5 were harvested. Cell pellets were washed with 10-mM HEPES, pH 7.4, containing 0.5-mM EDTA, followed by two washes with 10-mM HEPES. Aliquots were taken to determine viable counts. Cell pellet were digested with nitric acid at 100°C for an hour. Cu, iron, manganese, and zinc levels were determined by ICP-MS (8800 Triple Quadrupole, Agilent Technologies) by an operator blinded to treatment groups. Single quadrupole mode was used in all determinations, and a collision/reaction cell pressurized with helium was used to minimize potential spectral interferences. The concentration of trace elements was normalized to picogram per million CFU of *E. coli*.

### CpxR purification

*cpxR* was cloned into NdeI and BamHI sites of pET28a to generate pET28a_*cpxR*. This construct was transformed into *E. coli* BL21 and grown to log phase in LB at 37°C prior to addition of 0.5-mM IPTG and overnight incubation at 30°C. Cell pellets were lysed by sonication. CpxR was purified by using Ni-NTA column and dialyzed in chambers with 10-kDa MWCO membrane. Dialysis buffer contained 20-mM Tris-HCl, 150-mM NaCl, and 10% glycerol. Concentration of purified CpxR was determined by BCA assay. Purity of CpxR and wash fractions was verified by SDS-PAGE with Coomassie blue staining.

### EMSA

*yhiM* promoter region (0 to −400 bp) was amplified by PCR and biotinylated (Biotin 3′ End DNA Labeling Kit, Thermo Scientific). CpxR protein was phosphorylated by incubating with 25-mM acetyl phosphate at room temperature for 1 h just prior to use. CpxR protein at various concentrations (0 to 5 µg) was mixed with 40 ng/mL of biotin-labeled *yhiM* promoter in a binding reaction solution (LightShift Chemiluminescent EMSA Kit, ThermoScientific). The binding reaction was incubated at room temperature for 30 minutes, followed by addition of loading dye. Samples were electrophoresed on 6% polyacrylamide gels and transferred to nylon membranes. Membranes were cross-linked, and biotin-labeled DNA was detected by chemiluminescence (LightShift Chemiluminescent EMSA Kit, Thermo Scientific) in a ChemiDoc system (Bio-Rad).

### Mouse infection

Experimental UTI was induced in adult female CBA/J mice (4–6 weeks old, Jackson), as described previously (62, 63). Briefly, 10^8^ CFU of 1:1 mixture containing wild-type UPEC strain CFT073 and *ΔyhiM* mutant was instilled in the urinary bladders (*n* = 5–8 mice/group, repeated twice). Cultures of UPEC strains were incubated with shaking as described earlier in Materials and Methods. Urine samples were collected, and mice were euthanized at 48 h post-inoculation. Organs were removed aseptically, homogenized, plated on LB agar with or without kanamycin, and incubated aerobically at 37°C. Mutant strain grows on both plain and kanamycin-containing agar, whereas wild-type strain grows only on plain agar. Viable counts of wild-type and mutant bacteria were enumerated to calculate competitive indices (CI). CI = urine or tissue (mutant CFU/mL or g/wild-type CFU/mL or g) / inoculum (mutant CFU/mL/wild-type CFU/mL). CI of <1 indicates a fitness defect in the mutant, relative to its parental strain.

## SUPPLEMENTAL MATERIAL

Supplemental Material mbio.03522-23-s0001.pdf Supplemental table and figures.

ASM does not own the copyrights to Supplemental Material that may be linked to, or accessed through, an article. The authors have granted ASM a non-exclusive, world-wide license to publish the Supplemental Material files. Please contact the corresponding author directly for reuse.
