# Supplementary material for: The inner membrane protein YhiM links copper and CpxAR envelope stress responses in uropathogenic E. coli
Source: mBio. 2024 Mar 12;15(4):e03522-23. doi: 10.1128/mbio.03522-23 (PMC11005409; doi:10.1128/mbio.03522-23)
Supplement: Supplemental Material — Supplemental table and figures. [file mbio.03522-23-s0001.pdf]

SUPPORTING INFORMATION

Table S1. Oligonucleotide primers (5’-3’) used in this study

Mutant construction by Lambda Red recombination

P375 *yhiM* F TAACTGAAAAGACGAATCATATGTTTTTCGAAGTGTAGCAGAAAATATATGTGTAGGCTGGAGCTGCTTC

P376 *yhiM* R CTGAATAAAACCAGATAAGGCGCCCAACGCCTTATCTGAAGCCTTTTTAATGGGAATTAGCCATGGTCC

P391 *cpxA* F CCGTGGTTTAAAACCCTGCGTGGTCGCGGCTATCTGATGGTTTCTGCTTC GTGTAGGCTGGAGCTGCTTC

P392 *cpxA* R CAAATGCCGGATGCGGCGTAAACGCCTTATCCTGCCTACAAATGCGGAGTATGGGAATTAGCCATGGTCC

P511 *cpxR* F TGGATTAGCGACGTCTGATGACGTAATTTCTGCCTCGGAGGTATTTAAACAGTGTAGGCTGGAGCTGCTTC

P512 *cpxR* R GCGCCAGCGTCAGCCAGAAGATGGCGAAGATGCGCGGTTAAGCTGCCTAATGGGAATTAGCCATGGTCC

P603 *nlpE* F GGCGATGCGCGGCAAAGTGCGCAGCGGTCGGGAATAAAAAGAAGGAATGGGTGTAGGCTGGAGCTGCTTC

P604 *nlpE* R TAAGCGCAGCGCATGAGGCAATATTTCATTTGTTTCTGTCTCAAGACGGG ATGGGAATTAGCCATGGTCC

Mutant verification

P75 internal Km F	ACAACAGACAATCGGCTGCTCTGATGC
P61 Km F	GTGTAGGCTGGAGCTGCTTC
P62 Km R	ATGGGAATTAGCCATGGTCC
P377 y*hiM* F	GATGGCATCTTATAGACA
P378 *yhiM* R	GGTGGCAGAATAAGAAGC
P393 *cpxA* F	TGCACATTTCCAACCTGC

P394 *cpxA* R	GGAGTGTAGGCCTGATAA
P513 *cpxR* F	GCTCCCAAAATCTTTCTGTCG
P514 *cpxR* R	GCTGCTCAATCATCAGACCCT
P605 *nlpE* F	TGCATCGAAAGAGCGCAGGCT
P606 *nlpE* R	CCGGCCTACAAAATCGTGCTA

qPCR

P250 *copA* F	GCTACTGCTTTCCCGTCAAC
P250 *copA* R	TGACCAACAGGTCGATACCA
P293 *gapA* F	AAGTTGGTGTTGACGTTGTCGC
P294 *gapA* R	AGCGCCTTTAACGAACATCG
P449 *yhiM* F	GGTTATGCCGGTTCTGCTACA
P450 *yhiM* R	CAATCATGCCAACGCCGAAA
P457 *cpxA* F	TTGGCCTGGAGTCTGGCAAAA
P458 *cpxA* R	TCTGGTTAAAACTGGCACCGG
P515 *cpxR* F	AAAGCACTTCGCCAGACACAC
P516 *cpxR* R	TTCGGGAGATAGTCATCTGCG
P529 *fimA* F	ACCAGCTCTGCTGTCGGTTTT
P530 *fimA* R	TGTACCTAAGAAGGCAACAGC
P531 *fimD* F	GCACAATTGGCCTCAGATGAC
p532 *fimD* R	TAGTGACCTGTGCAGTACCAC
P533 *cpxP* F	CGAGATCTTATGCAACAGGCC

P534 *cpxP* R CGCGCACAGCGTTTTCATCAA

P535 *cusC* F GCGCAATTGCAAATAGCCGAA

P536 *cusC* R CTGTTCCAGCGCCAGAACATT

P575 *cueO* F GGTTGGTTTGGCGATACGTTG

P576 *cueO* R GAAATTGAGCGAGCGGGCATT

Complementation

P411 *yhiM* F CGGGTAGTAATGTTGTCATCA

P412 *yhiM* R CAACGCCTTATCTGAAGCCTT

EMSA

P611 *yhiM* F GACTATCACATAGCGT

P612 *yhiM* R ACCCATCAGTAAAGGA

Detection of transposon insertion sites

P319 KAN-2 F ACCTACAACAAAGCTCTCATCAACC

P320 R6KAN-2 R CTACCCTGTGGAACACCTACATCT

Invertible element PCR

P363 IE-PCR-F AGTAATGCTGCTCGTTTTGC

P364 IE-PCR-R GACAGAGCCGACAGAACAAC

F, forward; R, reverse.

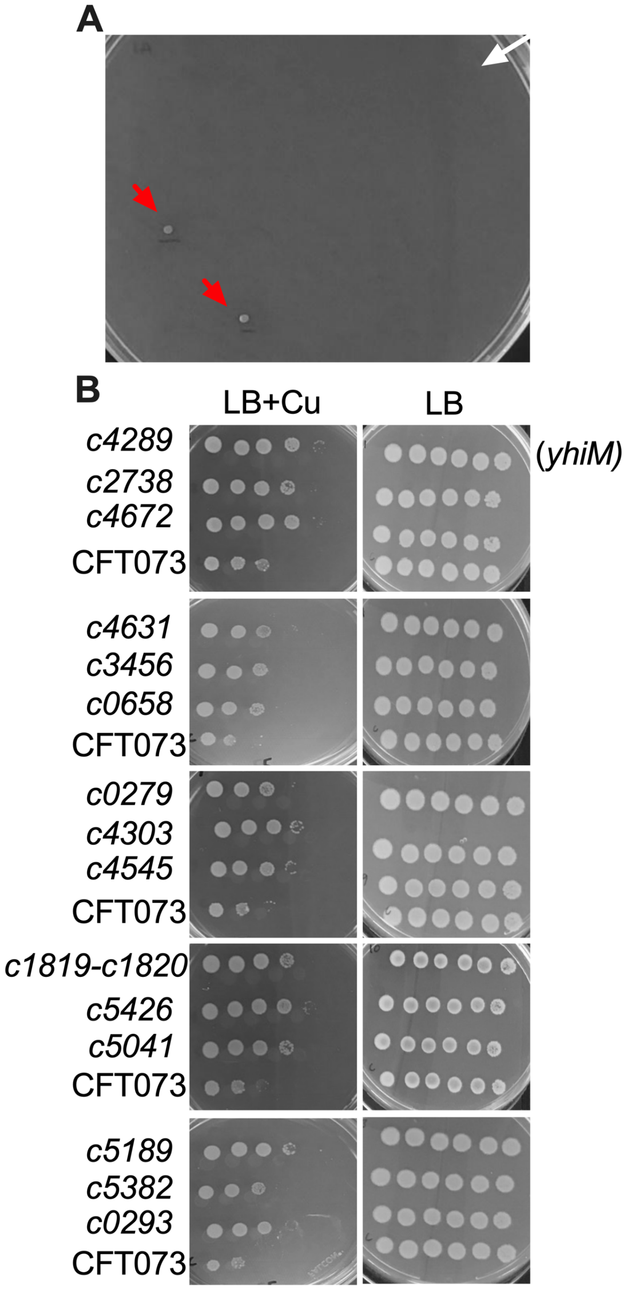

Fig. S1. Screening and verification of Tn*5* mutants with increased resistance to Cu. (A) Wild-type UPEC CFT073 (white arrow), and Tn*5* mutants were spot plated on Cu-containing LB plates to detect mutants with increased resistance to Cu (red arrows). (B) Cu resistance phenotype was further verified by dilution and spot plating on media with and without Cu. Genes interrupted by the transposon are indicated.

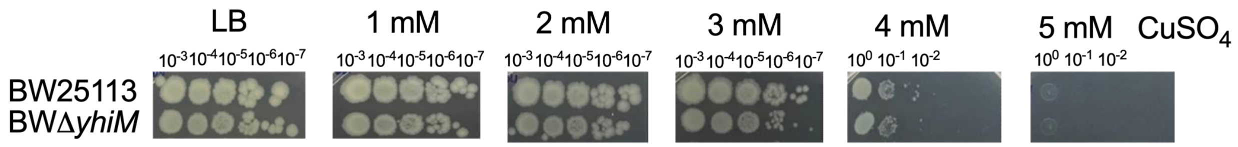

Fig. S2. YhiM is not involved in Cu resistance in *E. coli* BW25113. Wild-type and mutant strains were evaluated for Cu resistance. Dilutions that were spot plated are indicated and a representative image is depicted here.

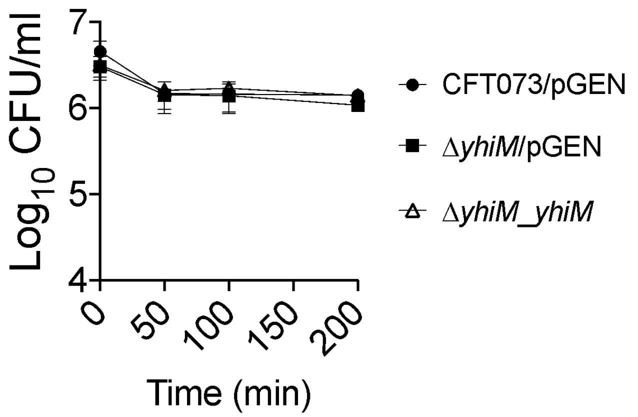

Fig. S3. YhiM does not affect survival of UPEC in acidic pH. Overnight cultures of wild-type, mutant and complemented mutant strains were diluted 1:100 in fresh LB (pH 2.5), and viable counts were determined at indicated time points. The experiment was repeated three times independently.

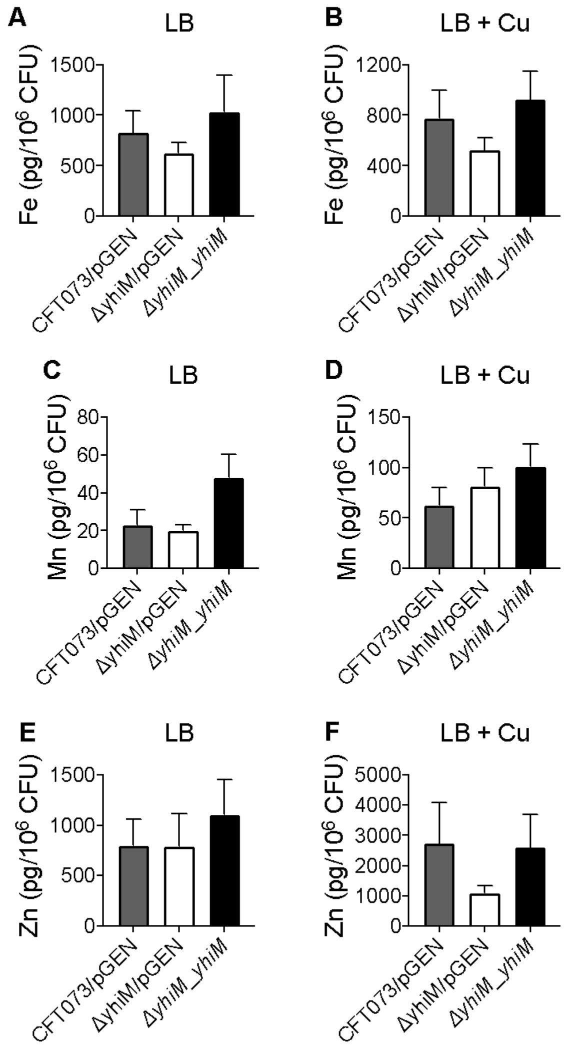

Fig. S4. Content of key transition metals in wild-type and *ΔyhiM* mutant and complemented mutant strains during Cu stress. (A-F) Cell-associated levels of Fe (A & B), Mn (C & D), and Zn (E & F) were determined in UPEC cultured in LB (A, C & E), or LB with 2 mM $CuSO_4$ (B, D & F) to mid-exponential phase (OD_{600}=0.5) by ICP-MS, and normalized to viable counts. Bars depict mean+SEM from three independent experiments. pGEN, empty vector.

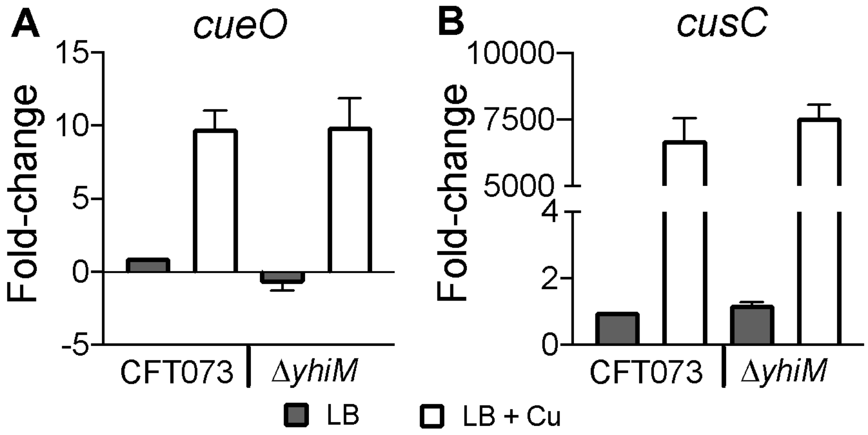

Fig. S5. Loss of YhiM does not affect *cueO* and *cusC* transcript levels. Expression of *cueO* (A) and *cusC* (B) transcript levels were quantified in wild-type and *ΔyhiM* mutant cultured in LB with or without 0.5 mM $CuSO_4$ by real-time PCR. Strains were cultured in LB to mid-logarithmic phase (OD_{600} = 0.5) before exposure to Cu for 20 minutes. Transcript levels were normalized to *gapA,* and relative expression was calculated. Bars depict mean+SEM from three independent experiments.

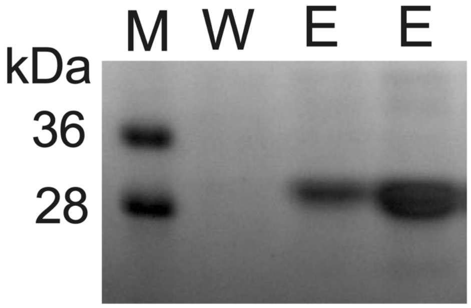

Fig. S6. CpxR overexpression and purification. CpxR was expressed in *E. coli* BL21 from an IPTG-inducible promoter in pET28a plasmid. Cell lysates were incubated with Ni-NTA, washed and CpxR was eluated. Wash and eluate fractions were separated on SDS-PAGE gels and stained with Coomassie blue. M, molecular mass marker; W, wash flow through; E, eluate.

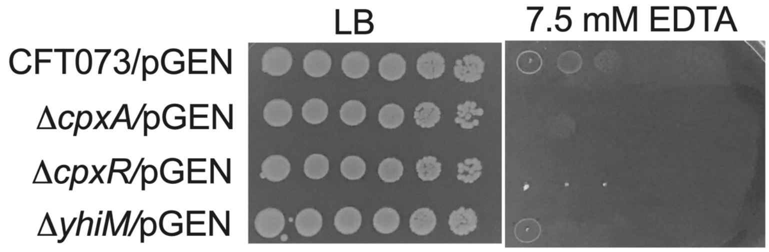

Fig. S7. CpxRA is required for growth during envelope stress. Wild-type and indicated mutant strains harboring an empty vector (pGEN) were diluted, and spot plated on LB agar with or without EDTA. A representative image acquired after 24 hours of growth from three independent experiments is presented here.

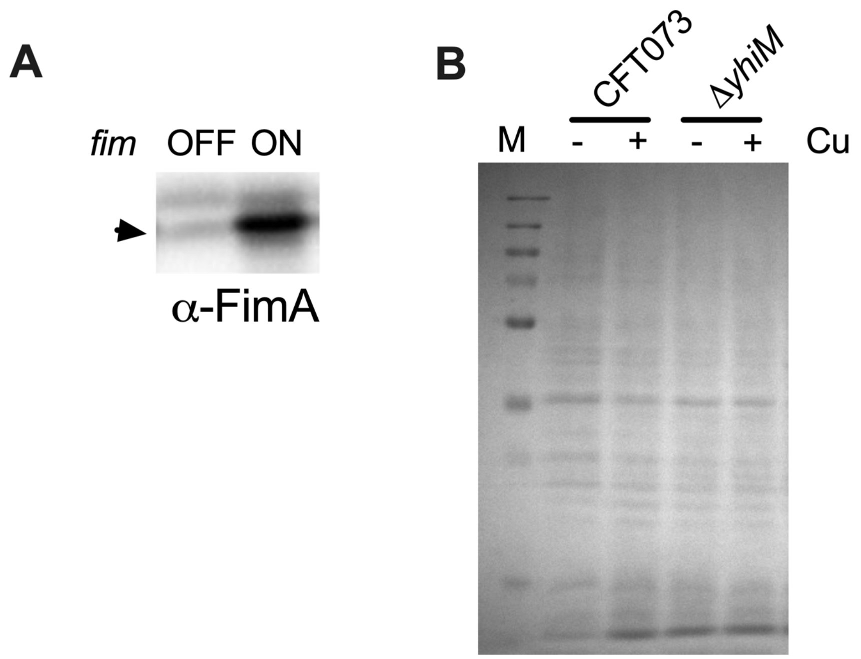

Fig. S8. Validation of FimA Immunoblotting and loading control. (A) Mutants with the *fim* promoter in the OFF and ON orientation were used as negative and positive controls for the Western blot. (B) Samples were electrophoresed in duplicate prior to Western blotting and a matching gel was stained with Coomassie blue to serve as a loading control.

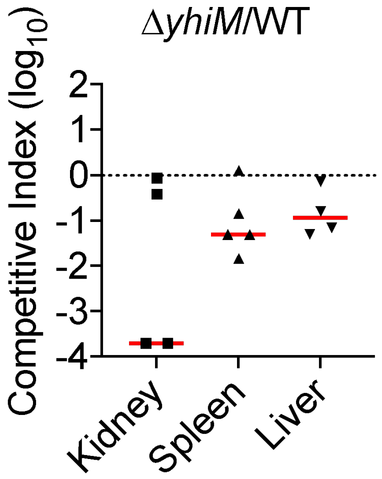

Fig. S9. Role of YhiM in UPEC fitness in a mouse model of UTI. Female mice were inoculated with a 1:1 mixture of wild-type strain and *ΔyhiM* mutant in the urinary bladder. Competitive indices were calculated as the ratio of mutant to wild-type strain *in vivo*, normalized to their ratio in the inoculum. Each symbol corresponds to results from a mouse, and bars indicate median. Dotted line, no loss of fitness in the mutant relative to wild-type strain (competitive index of 1).
